# Characteristics of female sexual dysfunctions and obstetric complications related to female genital mutilation in Omdurman maternity hospital, Sudan

**DOI:** 10.1186/s12978-017-0442-y

**Published:** 2018-01-08

**Authors:** Khalid Yassin, Hadeel A. Idris, AbdelAziem A. Ali

**Affiliations:** 1 Faculty of Medicine, Alnelain University, Khartoum, Sudan; 2 Federal Ministry of Health, Khartoum, Sudan; 3 Department of Obstetrics and Gynecology, Faculty of Medicine, Kassala University, Kassala, Sudan

**Keywords:** Female genital mutilation, Circumcision, Women health, Sudan

## Abstract

**Background:**

Female genital mutilation (FGM) is a major public health problem, especially in developing countries.

**Method:**

This was a prospective observational cohort study conducted over six months duration (1st July-31st December 2015) at Omdurman Maternity Hospital, Khartoum, Sudan, primarily to determine whether exposure to FGM/C (exposed Vs. non-exposed) and degree of exposure (type III Vs. type I) are associated with impaired sexual function or not?. As secondary objective, the study also investigated the association between FGM/C and postpartum complications (eg: difficulties in cervical examination, episiotomy wound infection, postpartum bleeding) by following the participants from the time of admission at the hospital, through vaginal delivery and until the 6th post-partum week.

**Results:**

A total of 230 (subjected to FGM/C) and 190 (not subjected to FGM/C) women were approached. The clinical examinations evidenced that the majority (67.8%) had FGM type *3*, while the remainder (32.2%) had type 1. The most common reported sexual complication was dyspareunia (76%). Bleeding following first attempt of sexual intercourse was reported in 35.2% followed by reduced sexual desire 62.6%, reduced sexual satisfaction 40.9% and need for surgery to release labial adhesions at first attempt of sexual intercourse 30.4%. With regard to FGM- related complications that occurred during labor 76.5% required an episiotomy, 61.7% experienced difficulties in cervical examination, 57.8% needed defibulations during second stage of labor, 26.5% complicated by episiotomy wound infection and 2.2% developed obstetric hemorrhage. In this study FGM/C was a significant factor increasing the risk of sexual complications. Interestingly when using logistic regression analysis the FGM-related complications were not significantly varied by FGM/C types.

**Conclusion:**

Our observations indicate that FGM/C is a serious public health problem and there should be an urgent intervention such as planned health education campaigns to end FGM/C practice.

## Plain English summary

Female genital mutilation (FGM) is a major public health problem, especially in developing countries. It is a dangerous practice that leads to many serious complications including hemorrhage, sepsis, severe pain, sexual dysfunction, primary infertility and obstetric complications. This study investigated whether exposure to FGM/C and degree of exposure are associated with impaired sexual function or not?. It also investigated the association between FGM/C and postpartum complications by following the participants from the time of admission at the hospital, through vaginal delivery and until the 6th post-partum week. After taking informed written consent, structured questionnaires were administered in local language (Arabic) using closed-ended questions to gather the different variable from 420 primigravida in a labor ward of the largest maternity hospital in Sudan. The finding of this study showed that the FGM is strongly associated with sexual and obstetric complications. Another key finding of this study is that all the forms of FGM are responsible for a high percentage of sexual complications. This study indicates that FGM constitutes a major public health problem and subjecting the women and the girls to several serious health problems and there should be an urgent intervention such as planned health education campaigns to end FGM practice.

## Background

Female genital mutilation (FGM) is a major public health problem, especially in developing countries [1]. Female genital mutilation is a cultural and traditional procedure and it is considered as a violation of the human rights of girls and women because of its negative impact on women health [2, 3]. Worldwide 200 million girls in 30 countries have undergone FGM and live with its health complications [4]. The procedure is outlawed in many countries [5, 6]. In Sudan girls typically undergo FGM between the age of 6 and 12 years and the cutting is always performed by the midwives without anesthesia, antibiotics or even sterilization, putting the girls in short and long term very serious consequences [7]. Female genital mutilation is highly prevalent in Sudan, it is reported in 83.3% among school girls and strongly associated with education [8], therefore the current study is directed to investigate the health complications associated with FGM and it is expected to provide the health planners and stakeholders with fundamental data for the development of strategic plan such as health education and good partnership towards the elimination of this practice.

## Methods

### Study design and study area

This was a prospective observational cohort study aimed, primarily, to determine whether exposure to FGM/C (exposed Vs. non-exposed) and degree of exposure (type III Vs. type I) are associated with impaired sexual function or not?. As secondary objective the study also investigated the association between FGM/C and postpartum complications (eg: difficulties in cervical examination, episiotomy wound infection, postpartum bleeding) by following the participants from the time of admission at the hospital, through vaginal delivery and until the 6th post-partum week. The study conducted over six months duration (1st July-31st December 2015) at Omdurman Maternity Hospital, Khartoum, Sudan which is the largest maternity hospital in Sudan established in 1957 and provide comprehensive obstetric and gynecological services to the public.

### Participants

All primigravida (subjected and not subjected to FGM/C) who experienced vaginal delivery during the study period were invited to participate in this study. There was a 100% participation rate and participants were adequately informed about consent to participate and they were given the option of refusing to participate during the informed consent process. Structured self-administered questionnaires were administered in local language (Arabic) using closed-ended questions (yes and no) to gather socio-demographic data (age, residence, education and occupation) and sexual function history (dyspareunia, bleeding following first intercourse, need surgery to release labial adhesions at first sexual intercourse, reduced sexual desire and sexual satisfaction) (An additional movie file shows this in more detail see Additional file 1). Complications that occurred during labor (difficulties in cervical examination, need for performing episiotomy, defibulations during second stage of labor and obstetric hemorrhage) were obtained from the participant’s clinical record. The types of FGM/C have been verified by physical examination. Ten female registrars in Obstetrics and Gynecology were selected to conduct the examination in the labor ward, they had been trained in relationship building, local cultures, believes, privacy, confidentiality, FGM/C typology and how to assist the respondents to fill the questionnaires. We used our own questionnaire which was constructed by the authors to consider different individual issues specific to our environment such as bleeding and need for surgery to release labial adhesion at time of first sexual intercourse, this is because the Female Sexual Function Index (FSFI) questionnaire was developed for the specific purpose of assessing domains of sexual functioning (e.g. sexual arousal, orgasm, satisfaction, pain). The questionnaire was revised by expert personnel in FGM/C, women health and right. We conducted one day session in which we discussed and approved the content of the questionnaire. This session was conducted by the authors and the expertise prior to the proposal approval. In this study the different sexual items were well explained for the respondents in local language. All women were followed for 6 weeks postpartum to report any complications happened (episiotomy wound infection). In this study we chose all the primigravida without exclusion criteria. Also we asked the women about whether the FGM/C is harmful or not and would they going to expose their daughter to the practice or not?. All women were examined to report the type of FGM. Female genital mutilation was defined according to the World Health Organization as all procedures involving partial or total removal of the external female genitalia or other injury to the female organs whether for cultural or other non-therapeutic reasons [9]. Again in this study we classified FGM according to WHO into four types: type1, also known as clitoridectomy: involves partial or total removal of the clitoris and/or prepuce; type2: involves partial or total removal of the clitoris and labia minora, with or without excision of the labia majora; type3: also known as infibulation, it entails removing part or all of the external genitalia and narrowing the vaginal orifice by re-approximating the labia minora and/or labia majora; type4: includes any form of other harm done to the female genitalia by pricking, piercing, cutting, scraping or burning [10].

### Data analysis

Data were entered into a computer database and SPSS software (SPSS Inc., Chicago, IL, USA, version 21.0), double checked, cleaned and verified by statistician before analysis. Means and proportions for the socio-demographic characteristics were compared between the groups of the study using student *t* and *×*2 test, respectively and *P* < 0.05 was considered significant. Logistic regression analysis was performed separately for dyspareunia, bleeding at first sexual attempt, reduced sexual desire, reduced sexual satisfaction and surgery to release labial adhesion to evaluate the effects of FGM\C on sexual function. Female genital mutilation was the independent variable and the sexual and obstetric complications were dependent variables. Confidence intervals of 95% were calculated and *P* < 0.05 was considered significant.

## Results

### Participant characteristics

A total of 420 primigravida delivered at Omdurman Maternity hospital were approached during this study, 230 were subjected to FGM/C and 190 were not subjected to FGM/C. There were no significant differences between the FGM/C group and non FGM/C group on socio-demographic characteristics. The mean age (SD) was not significantly different between the subjected to FGM/C group and those who were not, 27.2 (5.8) Vs. 26.0 (5.7), *P* = 0.1. The majority of the group who was subjected to FGM/C in comparison to the non-subjected to FGM/C group were of rural residence (61.7% Vs. 62.1%, *P* = 0.371), educated (57% Vs. 51.1%, *P* = 0.550) and housewives (84.7% Vs. 79.1%, *P* = 0.371), Table 1.

**Table 1:** Basic characteristic of the women “subjected and not subjected” to FGM/C′ and admitted to the labor ward at Omdurman Maternity Hospital, Sudan, 2015

Variable	Subjected to FGM/C (*n = 230*)	Not subjected to (*n = 190*)	*P* value
Age	27.2 (5.8)	26 (5.7)	0.100
Rural residence	142(61.7)	118 (62.1)	0.371
Illiteracy	99 (43)	93 (48.9)	0.550
Occupation, housewife	195 (84.7%)	151 (79.1)	0.371

Data was shown as number (%) as applicable

### FGM types and attitude

The clinical examinations evidenced that the majority had FGM type 3 (156/230, 67.8%), while the remainder (74/230, 32.2%) had type 1. The vast majority of the women in the subjected to FGM/C group (76.5%, 176/230) mentioned that they are not going to expose their daughter for FGM in the future and 65% (150/230) of them affirmed that FGM is a harmful practice. There was no differences between women with FGM/C and without FGM/C in plans to subject their daughters to FGM/C (76.5%% Vs. 74.8%, *P* = 0.411).

### Identified sexual complications

The majority (76.9%, *n* = 177) of women who have been subjected to FGM/C reported sexual complications. One hundred seventy seven women (76.9%) among them claimed that they had dyspareunia. Bleeding following first attempt of sexual intercourse was reported in 35.2% while reduced sexual desire, reduced sexual satisfaction and need for surgery to release labial adhesions at first attempt of sexual intercourse was reported in 62.6%, 40.9% and 30.4% respectively. Significantly more women in the group who have been subjected to FGM/C claimed that they have dyspareunia (76.9%% Vs. 46.2%, *P* = 0.001), reduced sexual desire (62.6%% Vs. 20%, *P* = 0.004) and reduced sexual satisfaction (40.9%% Vs. 6.3%, *P* = 0.002) compared with the other group, Table 2. Interestingly when using logistic regression analysis the FGM-related sexual complications were not significantly varied by FGM types, Tables 2 and 3.

**Table 2:** Distribution of sexual and obstetric complications among primigravida ‘subjected to FGM/C’ and ‘not subjected to FGM/C’ and admitted to the labor ward at Omdurman Maternity Hospital, Sudan, 2015

Complication	Subjected to FGM/C (*n = 230*)	Not subjected to FGM/C (*n = 190*)	*P* value
Dyspareunia	177 (76.9%)	46 (24.2%)	0.001
Bleeding at first sexual attempt	81 (35.2%)	3 (1.5%)	<0.001
Reduced sexual desire	144 (62.6%)	38 (20%)	0.004
Reduced sexual satisfaction	94 (40.9%)	12 (6.3%%)	0.002
Surgery to release labial adhesion	70 (30.4%)	00 (00%)	<0.001
Difficulty in pelvic examination	142 (61.7%)	35 (18.4.%)	0.021
Episiotomy	176 (76.5%)	57 (30%)	0.011
Deinfibulation	133 (57.8%)	0 (00)	<0.001
Infection of episiotomy	61 (26.5%)	2 (1.1%)	<0.001
Hemorrhage	5 (2.2%)	3 (1.5%)	0.275

Data was shown as number (%) as applicable

**Table 3:** Corrected odds ratio and 95% confidence interval for subjected women to FGM/C and those who were not subjected to FGM/C to evaluate the effects of FGM\C on sexual function (dyspareunia, bleeding at first sexual attempt, reduced sexual desire, reduced sexual satisfaction and surgery to release labial adhesion) in Omdurman Maternity Hospital, Sudan, 2015

Variables	Subjected to FGM/C		Not subjected to FGM/C	
	OR (95% CI)	*P*	OR (95% CI)	*P*
Dyspareunia	1.0 (0.9–1.0)	0.6	0.9 (0.9–1.0)	0.1
Bleeding at first sexual attempt	1.1 (1.0–1.1)	0.2	1.0 (1.0–1.2)	0.2
Reduced sexual desire	1.4 (1.0–1.2)	0.3	1.3 (0.7–2.4)	<0.2
Reduced sexual satisfaction	1.1 (0.9–1.0)	0.1	1.0 (1.0–1.1)	0.5
Surgery to release labial adhesion	1.5 (0.9–1.2)	0.6	1.2 (0.8–2.0)	0.2

### Postpartum complications

With regard to FGM- related complications that occurred during labor 76.5% required an episiotomy, 61.7% experienced difficulties in cervical examination, 57.8% needed defibulations during second stage of labor, 26.5% complicated by episiotomy wound infection and 2.2% developed obstetric hemorrhage. More proportion of the women among those who have been subjected to FGM/C had difficulties in cervical examination (61.7% Vs. 18.4%), required an episiotomy (76.5% Vs. 30%), needed defibulations during second stage of labor (57.8% Vs. 00) and developed episiotomy wound infection (26.5% Vs. 1.1%), Table 2.

## Discussion

The finding of this study showed that the FGM is strongly associated with sexual and obstetric complications. Another key finding of this study is that all the forms of FGM are responsible for a high percentage of complications. This study indicates that FGM constitutes a major public health problem subjecting the women and girls to several serious health problems. In the present study dyspareunia (pain feel during sexual intercourse) was reported in 76.9% among the group subjected to FGM/C, while less than quarter (24.2%) of the other group reported dyspareunia. The FGM related health consequences have been investigated and reported. A more recent systematic review showing that women with FGM were more likely to experience dyspareunia, reduced sexual satisfaction and reduced sexual desire [11, 12]. Our result is in agreement with study which was conducted in Egypt, where the authors found that women with FGM have higher rates of dyspareunia and lack of sexual desire [13]. This could be explained by the fibrosis and rigid scar tissue following FGM which predispose to narrowing of the vaginal orifice and muscular spasm that make intercourse painful and difficult. These physical factors again contributed to psychological factor, where the painful sexual practice will drive women to lose both sexual desire and satisfaction. It might be difficult in the present time to reduce these risk and complications, this is because there is lack of knowledge regarding health consequences associated with FGM, even among local health professionals like nurses or midwifes in Sudan [14]. In this study difficulty in cervical examination during labor was reported in more than half (61.7%) of women who have been subjected to FGM/C, so pelvic examination on infibulated women can be challenging. The narrow introitus can made bimanual examination difficult, and if not impossible. Again this findings are supported by *De Silva* [15]. In the present study 76.5% of the women with FGM required an episiotomy and this is highly justifiable because performance of episiotomy may limit the degree of perineal laceration. About 57.8% of the women with FGM needed defibulations during second stage of labor to reduce the risks of delivery with infibulated scar which may lead to prolonged second stage of labor and increase risk of spontaneous laceration. Nearly one third (26.5%) of the women with FGM reported episiotomy wound infection. This possibly explained by the collection of blood and lochia behind the sutured skin and result in poor hygiene of the vagina and thus represent good media for the growth of the bacteria. Our study showed no association between obstetric hemorrhage and FGM and this is possibly explained by high incidence rate of performing episiotomy wound to prevent laceration and non linear tear during labor. This results is in contrast to the findings from six African countries including Sudan, where the authors reported that a loss of 130,000 life years is expected owing to FGM’s association with obstetric hemorrhage [16]. One of the strength of this study is the use of close ended question regarding the sexual satisfaction and the answers rely on the participant’s experience and feeling without scoring however we think one of the major limitation of this study is its inability to measure other sexual dysfunction items such as sexual arousal and orgasm. Again our results are limited by using a questionnaire instrument that has not been validated. Also we did not conduct separate analyses by type of FGM/C but rather just with FGM/C or without FGM/C.

## Conclusion

Our observations indicate that FGM is a serious public health problem and there should be an urgent intervention such as planned health education campaigns to end FGM practice.

## Additional file

Additional file 1: Variables included in the questionnaire. The data include basic charecteristics, FGM typology, sexual function history, complications during second stage of labor and attitudes towards FGM. (DOCX 17 kb)

## Abbreviations

CI: Confidence interval
FGM: Female genital mutilation
OR: Odds ratio
*P*: *P*- value
SD: Standard deviation
SPSS: Statistical Package for the Social Sciences
WHO: World Health Organization
