# Supplementary material for: Characteristics of female sexual dysfunctions and obstetric complications related to female genital mutilation in Omdurman maternity hospital, Sudan
Source: Reprod Health. 2018 Jan 8;15:7. doi: 10.1186/s12978-017-0442-y (PMC5759286; doi:10.1186/s12978-017-0442-y)
Supplement: Additional file 1: — Variables included in the questionnaire. The data include basic charecteristics, FGM typology, sexual function history, complications during second stage of labor and attitudes towards FGM. (DOCX 17 kb) [file 12978_2017_442_MOESM1_ESM.docx]

**Characteristics of female sexual dysfunctions and obstetric complications related to female genital mutilation in Omdurman maternity hospital, Sudan**

**Introduction**

**We are a group of researchers (Prof Khalid Yassin (Alneelain University), Prof. AbdelAziem A. Ali (Kassala University) and Dr. Hadeel A. Idris (Fedral Minsitry of Health) concern about the women and public health in Sudan and we have many works about the Female Genital Cutting (FGM/C), Child Marriage as well as obstetric problems. WELCOME AND THANKS YOU for response to participate in this study. This questionnaire is designed to assess the impact of FGM/C on sexual health and obstetric outcome among Sudanese women. And by answering our questions; you will provide information that ultimately improve the quality of care and women health. It will take about 20 minutes to be filled. Please note that your information IS NOT personally identifiable and all responses are CONFIDENTIAL. In this questionnaire there is a part dealing with the type of the FGM which is going to be determined by a female doctor. Don't hesitate if you have any question.**

**Section 1: General Characteristics**

**Serial N:**…………………………………………………………………………

**Unit: ………………………….. File Number:**

**Primary phone N: …………………Secondary phone N……………………..**

**Age:** ………………**Residence:** Rural Urban

**Occupation**: housewife employee Skilled worker Non skilled worker

**Education**: Illiterate Non formal < Secondary ≥ Secondary

**Section 2: FGM/C Type (For the doctor):** type1,: involves partial or total removal of the clitoris and/or prepuce; type2: involves partial or total removal of the clitoris and labia minora, with or without excision of the labia majora; type3: it entails removing part or all of the external genitalia and narrowing the vaginal orifice by re-approximating the labia minora and/or labia majora; type4: includes any form of other harm done to the female genitalia by pricking, piercing, cutting, scraping or burning

**t**ype 1 type 2 type 3 type 4

**Section 3: Sexual Function History**

**Dyspareunia:** yes no **Bleeding following first sexual intercourse:** yes no **Need surgery to release labial adhesion at first sexual intercourse:** yes no **Reduced sexual desire :** yes no

**Section 4: Complications during 2^nd^ stage of labor and following labor:**

**Difficulty in cervical examination**: yes no

**Need for episiotomy:** yes no

**Defibulation during second stage of labor**: yes no

**Obstetric bleeding**: yes no

**Episiotomy wound infection**: yes no

**Section 5: Attitude towards FGM:**

**Is the FGM/C harmful**: yes no

**Are you going to exposed your daughter to this practice:** yes no
